# Chronic postsurgical pain: From risk factor identification to multidisciplinary management at the Toronto General Hospital Transitional Pain Service

**DOI:** 10.1080/24740527.2019.1574537

**Published:** 2019-07-30

**Authors:** Joel Katz, Aliza Z. Weinrib, Hance Clarke

**Affiliations:** a Pain Research Unit, Department of Anesthesia and Pain Management, Toronto General Hospital, Toronto, Ontario, Canada; b Department of Psychology, York University, Toronto, Ontario, Canada; c Department of Anesthesia, University of Toronto, Toronto, Ontario, Canada

**Keywords:** Transitional Pain Service, acceptance and commitment therapy, chronic postsurgical pain, risk factors, opioid use, opioid weaning, pain, pain interference

## Abstract

**Background**: Chronic postsurgical pain is a highly prevalent public health problem associated with substantial emotional, social, and economic costs.

**Aims**: (1) To review the major risk factors for chronic postsurgical pain (CPSP); (2) to describe the implementation of the Transitional Pain Service (TPS) at the Toronto General Hospital, a multiprofessional, multimodal preventive approach to CPSP involving intensive, perioperative psychological, physical, and pharmacological management aimed at preventing and treating the factors that increase the risk of CPSP and related disability; and (3) to present recent empirical evidence for the efficacy of the TPS.

**Methods**: The Toronto General Hospital TPS was specifically developed to target patients at high risk of developing CPSP. The major known risk factors for CPSP are perioperative pain, opioid use, and negative affect, including depression, anxiety, pain catastrophizing, and posttraumatic stress disorder–like symptoms. At-risk patients are identified early and provided comprehensive care by a multidisciplinary team consisting of pain physicians, advanced practice nurses, psychologists, and physical therapists.

**Results**: Preliminary results from two nonrandomized, clinical practice–based trials indicate that TPS treatment is associated with improvements in pain, pain interference, pain catastrophizing, symptoms of anxiety and depression, and opioid use. Almost half of opioid-naïve patients and one in four opioid-experienced patients were opioid free by the 6-month point.

**Conclusions**: These promising results suggest that the TPS benefits patients at risk of CPSP. A multicenter randomized controlled trial of the TPS in several Ontario hospitals is currently underway.

## Abstract (es)

**Contexte**: La douleur chronique postopératoire est un problème de santé publique très prévalent, associé à des coûts émotionnels, sociaux et économiques considérables.

**Buts**: (1) Étudier les facteurs de risque importants pour la douleur chronique postopératoire; (2) décrire la mise en œuvre du Service de la douleur transitoire à l’Hôpital général de Toronto, une approche préventive multiprofessionnelle, multimodale à la douleur chronique postopératoire fondée sur une prise en charge intensive, périopératoire, psychologique, physique et pharmacologique pour prévenir et traiter les facteurs qui augmentent le risque de douleur chronique postopératoire et l’incapacité qui s’y rattache; et (3) présenter des données probantes empiriques récentes en ce qui concerne l’efficacité du Service de douleur transitoire.

**Méthodes**: Le Service de la douleur transitoire de l’Hôpital général de Toronto a été expressément mis sur pied pour cibler les patients ayant un risque élevé de souffrir de douleur chronique postopératoire. Les principaux facteurs de risque connus pour la douleur chronique postopératoire sont la douleur périopératoire, la consommation d’opiacés et un affect négatif, y compris la dépression, l’anxiété, la catastrophisation de la douleur et les symptômes apparentés au syndrome de stress post-traumatique. Les patients à risque sont repérés de manière précoce et des soins complets leurs sont prodigués par une équipe multidisciplinaire composée de médecins spécialistes de la douleur, d’infirmières en pratique avancée, de psychologues et de physiothérapeutes.

**Résultats**: Les résultats préliminaires de deux essais cliniques non randomisés fondés sur la pratique indiquent que le traitement prodigué par le Service de la douleur transitoire est associé à des améliorations en ce qui concerne la douleur, l’interférence de la douleur, la catastrophisation de la douleur, les symptômes d’anxiété et de dépression, et la consommation d’opiacés. Près de la moitié des patients n’ayant jamais consommé d’opioïdes et un patient sur quatre parmi ceux qui avaient déjà consommé des opioïdes n’en consommaient plus après six mois.

**Conclusions**: Ces résultats prometteurs suggèrent que le Service de la douleur transitoire profite aux patients à risque de douleur chronique postopératoire. Un essai multicentre randomisé et contrôlé du Service de la douleur transitoire est actuellement en cours dans plusieurs hôpitaux ontariens.

Chronic pain is the silent epidemic of our times.^1^ It causes enormous human suffering and drains the Canadian economy and health care system of valuable resources. Health-related quality of life of Ontarians with chronic pain is lower than that reported by people with most other chronic diseases, including heart disease, diabetes, and chronic obstructive pulmonary disease.^2^ The annual incremental medical cost to manage chronic pain in Ontario is estimated at $1742 per person in 2014 Canadian dollars,^3^ which translates to a ~$10 billion burden annually to the Canadian health care system, not including direct out-of-pocket costs incurred by the person with pain or indirect costs such as lost income. Chronic postsurgical pain (CPSP) is a significant driver of this cost^4^ given the high rates of CPSP. The magnitude of the problem is evident when one jointly considers that 312 million major surgeries are performed annually worldwide^5^ and the one-year incidence of moderate-to-severe CPSP is ~12% and ~22% for adults^6^ and children,^7^ respectively. It is not surprising, then, that more than 20% of adults^8^ and 17% of children^9^ attending specialized chronic pain centers have been referred for CPSP. Innovative research designs^10–12^ and novel solutions^13,14^ are needed to halt—and potentially reverse—the transition of acute pain to chronic pain, improve quality of life, and reduce personal/system costs related to unnecessary hospitalizations, medications, disability, and unemployment.

The aims of the present article to are (1) review the major known risk factors for CPSP; (2) describe the implementation of the Transitional Pain Service (TPS) at the Toronto General Hospital, a multiprofessional, multimodal preventive approach to CPSP involving intensive, perioperative psychological, physical, and pharmacological management aimed at preventing and treating the factors that increase the risk of CPSP and related disability; and (3) present recent empirical evidence for the efficacy of the TPS.

## Chronic postsurgical pain—Definition

Substantial variability exists among surgical procedures, including the anatomic structures and physiologic processes affected as well as the time required to heal and recover. Nevertheless, the following 6-point definition appears to capture the most important aspects of CPSP.^15,16^ The pain (1) developed after a surgical procedure, (2) is at least 2 months in duration, (3) interferes significantly with health-related quality of life, (4) is a continuation of acute postsurgical pain or develops after an asymptomatic period, (5) is localized to the surgical field and/or projected to territory or dermatome innervated by a nerve in the surgical field and (6) is not caused by other factors (e.g., preoperative pain, recurrence after surgery for cancer, chronic infection have been ruled out).

## Risk factors for chronic postsurgical pain

Considerable progress has been made in identifying risk and protective factors for CPSP,^7,17–26^ although the question of causality remains unanswered.^19,27–29^ There are many risk factors for CPSP. The following are those most consistently reported in the literature. Type of surgery has long been recognized as a major risk factor for CPSP, with surgeries that involve deliberate or inadvertent damage to nerves showing the highest prevalence,^17,19^ though not all CPSP is neuropathic.^30^

The most robust risk factor for CPSP is pain itself.^19,31^ The data clearly show that the presence^32^ and intensity of preoperative chronic pain,^6,33–35^ the intensity of acute postoperative pain,^6,23,32,33,36^ the time spent in severe pain after surgery,^6^ pain intensity in the weeks after surgery,^35,37,38^ and pain in other body parts^32,34,39^ reliably predict CPSP across a range of surgical procedures. Higher consumption of postoperative analgesics, typically a proxy for intense postoperative pain, is associated with more intense CPSP.^38,40,41^ Negative cognitive–affective states including perioperative depression,^42,43^ anxiety,^25,32,35,43,44^ pain catastrophizing,^18,44^ and posttraumatic stress symptoms^45^ also predict the development of CPSP. Finally, preoperative opioid use, with a prevalence of ~25%,^46^ is a risk factor for CPSP,^32,47^ in part due to opioid-induced hyperalgesia.^48,49^

Negative affect and pain catastrophizing are also risk factors for intense, acute postoperative pain and excessive opioid use.^50–52^ Inadequately controlled acute pain and excessive opioid use delay recovery and hospital discharge after many surgeries.^53–56^ Notably, many of the above risk factors for CPSP are also associated with persistent opioid use after surgery, including mood disorders,^57^ anxiety,^57^ pain catastrophizing,^58^ preoperative neuropathic and nonneuropathic pain,^57^ and greater pain intensity on the day of surgery.^58^

Management of these known risks before and after surgery is hypothesized not only to reduce pain, suffering, and opioid misuse but also to benefit the health care system by facilitating earlier discharge and reducing costs.^59–61^ The following sections describe the Toronto General Hospital TPS and preliminary clinical outcomes.

## Toronto General Hospital Transitional Pain Service—Development and description

The Toronto General Hospital TPS^59–61^ was established in 2014 to address the problem of CPSP with a seamless approach to perioperative pain and opioid use using multidisciplinary, integrated care. Patients are assessed and managed as early as the preoperative visit, treatment is extended into the in-hospital setting after surgery, and it is maintained for up to 6 months across the post–hospital discharge period once patients have returned home. The primary aim of the TPS is to offer timely and effective treatment to patients at high risk of developing chronic postsurgical pain and persistent opioid use after undergoing a variety of surgical procedures, including those for cancer (e.g., thoracic, breast, gastrointestinal, head and neck), cardiac disease (e.g., coronary artery bypass graft, heart valve repair), and organ transplants (e.g., kidney, lung, liver, heart, pancreas). The three major goals of the TPS are to (1) provide comprehensive pre- and postoperative pain management for patients who are at high risk of developing chronic postsurgical pain and pain disability, (2) manage opioid medication while in hospital and after discharge, and (3) improve coping and functioning in the immediate and long term to provide as high a quality of life as possible. At present, clinical services at the TPS include multimodal medication optimization by anesthesiologists, postsurgical physical therapy and acupuncture, and a pain psychology intervention consisting of pain education, mindfulness training, brief hypnosis, and a form of cognitive–behavioral treatment called acceptance and commitment therapy (ACT). The service also includes an administrative assistant and a patient care coordinator. In 2016, the TPS began a partnership with ManagingLife, whose mobile platform and app, Manage My Pain, allows TPS patients to quickly and easily track their pain on a daily basis using an Apple iPhone, Android smartphone, or a responsively designed web app through their mobile or desktop browser.^62^

The TPS provides proactive, timely support in a multidisciplinary setting to inpatients and outpatients with complex postsurgical pain for up to 6 months after surgery. Patients are screened for physical and mental health problems and flagged for known risk factors (Table 1) if they have a history of anxiety, depression, high levels of pain catastrophizing, chronic opioid use, and/or preexisting chronic pain. Intensive intervention is provided to patients who are at the highest risk of developing CPSP and persistent, high-dose opioid use.

**Table 1.** Referral criteria for admission to the Transitional Pain Service.^a^

“Pain alert” patients
Presurgical chronic pain
History of drug abuse
Currently on opioid, methadone, or buprenorphine maintenance therapy
Severe postsurgical pain
Prolonged Acute Pain Service stay
Surgical patients with repeat Acute Pain Service consultation
Medically stable postsurgical patients with complex pain problems that prevent discharge
High postsurgical opioid consumption
Consumption of > 90 MME/day
Methadone or buprenorphine patients without a community pain specialist
Patients discharged with a prescription for a long-acting opioid
Interventional postsurgical procedures (e.g., stump catheters postamputation)
Emotional distress
Depression
Anxiety
Pain catastrophizing
Other psychosocial concern(s) identified by questionnaires or Acute Pain Service/Transitional Pain Service member

^a^Adapted with permission from Katz et al.^59^ MME = morphine milligram equivalents.

## Toronto General Hospital Transitional Pain Service—Implementation

### Psychology at the TPS

There is growing understanding of the important role that psychological interventions can play in reducing pain perception, negative affect, and avoidance behaviors after surgery.^29^ The main goal of the TPS ACT intervention is to teach patients a mindful approach to their postsurgical pain that allows them to live a fuller life.^29,63^ The ACT intervention teaches them how to stop the negative cycle of intense postoperative pain, emotional distress, behavioral avoidance, and escalating opioid use that inhibits functioning and degrades quality of life. Patients learn to expand their capacity to experience pain—including the negative thoughts and feelings that inevitably arise when pain is present. Pain sensations—as well as the patients’ reactions to them—are observed neutrally and nonreactively, while focusing on enhancing motivation and commitment to engage in personally meaningful, achievable, goal-oriented activities. They are taught to do so without engaging in problematic avoidance behaviors that typically make the pain worse and limit functioning. Ultimately, patients become more *psychologically flexible*; they learn to adapt their behavior in a way that enables them to live a rich and meaningful life while open to their internal psychological and emotional experiences, including pain.

### Other Disciplines at the TPS

In addition to our psychology team, the nursing, physiotherapy/acupuncture specialists, and recently our yoga specialist are all integral to the TPS. The Acute Pain Service nurse practitioners are the backbone of the TPS. They identify patients immediately after surgery who are not recovering as they should and then refer to our inpatient coordinator, who then arranges an in-hospital visit and the scheduling of the outpatient clinic visit. The outpatient clinic has also recently expanded to include a pain and opioid misuse (addiction) specialist nurse practitioner who has been a critical addition to the team and a significant resource to the hospital. The nurse practitioner’s role is to manage complex pain patients with opioid and other use disorders within the institution (expanding beyond perioperative care). Our acupuncturist manages patients who are amenable to treatment in the outpatient setting and we are planning to evaluate its effectiveness in the immediate postoperative time period. Finally, we have a certified hatha yoga instructor who has developed a postoperative yoga pathway that we are currently evaluating for efficacy.

### Challenges in the implementation of the TPS

We encountered several barriers to implementation of the TPS. Some have been overcome but we continue to grapple with others. First, institutional acceptance of the program took time, and acceptance by our surgical colleagues regarding the added value of this novel care pathway was initially lukewarm. Today, the referrals overwhelm the team and currently a major limitation is the inability to keep up with the increased capacity demands within the institution. Discharging patients after they have stabilized has also proven to be a challenge. This is partly due the patients’ positive connection to the interdisciplinary team and their reluctance to move back to a single-provider model of care. The lack of primary care providers for some patients has also proven to be a factor limiting our ability to discharge TPS patients within the 3- to 6-month postoperative time frame. The added burden of the opioid crisis has also delayed our ability to discharge stable TPS patients who were unable to wean off their opioid medications because many primary care providers simply refuse to inherit opioid prescriptions not initiated by them or refuse outright to prescribe opioids at all. We also encountered initial challenges integrating the Manage My Pain (MMP) app into the daily clinical service of the TPS. One of the biggest hurdles in the implementation of the MMP platform at Toronto General Hospital was safe stewardship of patient health information. Issues involved data security, sharing patient data with the care team, authenticating the clinical user, and access to patient data after TPS treatment has ended. These concerns regarding the safeguarding of patients’ electronic private health information have been resolved to satisfaction of the TPS, MMP, and patients.^62^

## Preliminary evaluation of the TPS

At present, the best evidence we have for the efficacy of the TPS comes from two clinical practice–based cohort studies^64,65^ and a detailed case report^63^ showing promising psychosocial outcomes and opioid weaning rates. The two clinical practice–based studies are reviewed in detail below. A multicenter randomized controlled trial of the TPS in several Ontario hospitals is currently underway, funded by the Ontario Ministry of Health and Long-Term Care.

### TPS ACT-based psychological outcomes

In the first study, patients receiving TPS psychological services (ACT group) were compared to those not receiving psychological services (no ACT group) on measures of pain, pain interference, key psychological constructs, and opioid use for the duration of TPS treatment.^64^

A total of 382 TPS patients participated in the study. Ninety-one received ACT and 252 did not (no ACT). Pain intensity, pain interference, sensitivity to pain traumatization,^66^ pain catastrophizing,^67^ symptoms of anxiety and depression,^68^ and opioid use were compared between the two groups and across time, beginning with the first TPS visit and ending with the last. Patients referred to the ACT group were significantly more likely to report a mental health condition preoperatively, had significantly higher opioid use at the first postsurgical visit, and, at both time points, reported significantly higher sensitivity to pain traumatization and anxiety scores than the no ACT group. These pretreatment differences are not surprising given that patients who are referred to TPS psychology services typically are the most distressed and are having the most difficulty coping. On the other hand, likely because of these differences, the ACT group was involved with the TPS for a significantly greater number of weeks and had significantly more medical visits to the TPS than the no ACT group.

By the last TPS visit, both groups showed significant reductions in pain intensity, pain interference, pain catastrophizing, anxiety, and opioid use. Compared to the no ACT group, the ACT group showed greater reductions in opioid use and pain interference. Moreover, only the ACT group showed a significant reduction in depressed mood by the last TPS visit. These results indicate that patients who are referred for psychology services upon admission to the TPS are a higher-risk group of patients. Nevertheless, learning the ACT approach to behavioral pain management enabled them to wean off opioid medications to a greater extent than the lower-risk, no ACT group while at the same time reporting greater improvements in mood and less pain interference. However, differences in treatment duration between the ACT and no ACT groups raise the question as to whether the no ACT group would have achieved additional reductions in opioid weaning, pain interference, and depressive symptomology given an equivalent follow-up time. With these caveats in mind, these results provide preliminary support for the ACT-based intervention in targeting and successfully managing TPS patients at risk of CPSP and persistent opioid use.

### TPS opioid consumption and opioid weaning rates

The second clinical practice–based study^65^ compared opioid consumption and opioid weaning rates an average of 6 months after surgery between TPS patients who were opioid naïve or opioid experienced presurgically. Opioid consumption in daily morphine milligram equivalents (MME/day) was examined on admission to the TPS, at hospital discharge after surgery, and on the day of their last TPS visit an average of 6 months after surgery.

**Figure 1.**
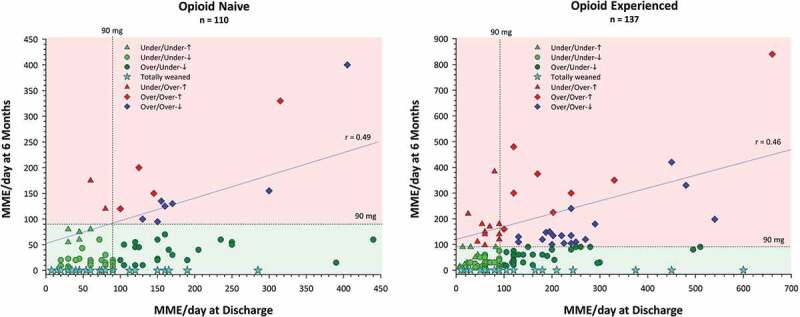
Mean daily opioid use in daily morphine milligram equivalents (MME/day) at the end of TPS treatment shown as a function of daily morphine milligram equivalents at hospital discharge (i.e., prior to TPS treatment) for opioid-naïve and opioid-experienced patients. Also shown is the 90 MME/day maximum dose recommended by the U.S.^69^ and Canadian^70^ opioid guidelines (dashed lines). Based on the 90 MME/day, patients in the two lower quadrants (green shading) represent treatment successes and those in the two upper quadrants (red shading) represent treatment failures. Green triangles represent patients who were under 90 MME/day on both occasions and higher at 6 months than at hospital discharge; green circles represent patients who were under 90 MME/day on both occasions and lower at 6 months than at hospital discharge; dark green circles represent patients who were over 90 MME/day at hospital discharge and under at 6 months; cyan stars represent patients who were totally weaned (MME/day = 0) by 6 months; red triangles represent patients who were under 90 MME/day at hospital discharge and over at 6 months; red diamonds represent patients who were over 90 MME/day on both occasions and higher at 6 months than at hospital discharge; blue diamonds represent patients who were over 90 MME/day on both occasions and lower at 6 months than at hospital discharge. Left panel adapted with permission from Clarke et al.^65.^

Opioid-experienced patients (*n* = 137) were taking a mean of 78.8 ± 100.2 MME/day prior to surgery. The daily dose almost doubled to 140.5 ± 124.0 MME/day by the time they were discharged from hospital after surgery. At the last TPS visit ~6 months after surgery they were taking 78.3 ± 113.9 MME/day (44.3% decrease), which was essentially the same dose they had been taking prior to surgery (Figure 1). In terms of weaning rates, by the 6-month mark, 35 opioid-experienced patients (25%) had been completely weaned, 50 (36%) had reduced their opioid use by >50% of the hospital discharge dose, 27 (19%) had reduced their opioid use by <50%, and 27 (19%) had increased opioid use since hospital discharge.

In contrast, opioid-naïve patients, who nonetheless were identified as high risk for postsurgical pain (*n* = 110), were taking a mean of 106.7 ± 80.6 MME/day at the time of hospital discharge after surgery, which was reduced to 37.3 ± 61.1 MME/day at the final TPS visit (65% decrease) ~6 months after surgery. In terms of weaning rates, by 6 months after surgery, 51 (46%) had been successfully weaned from opioids, 39 (35%) had reduced opioid use by >50% of their hospital discharge dose, 11 (10%) had reduced opioid use by <50%, and 9 (8%) had increased their opioid use from hospital discharge.

**Figure 2.**
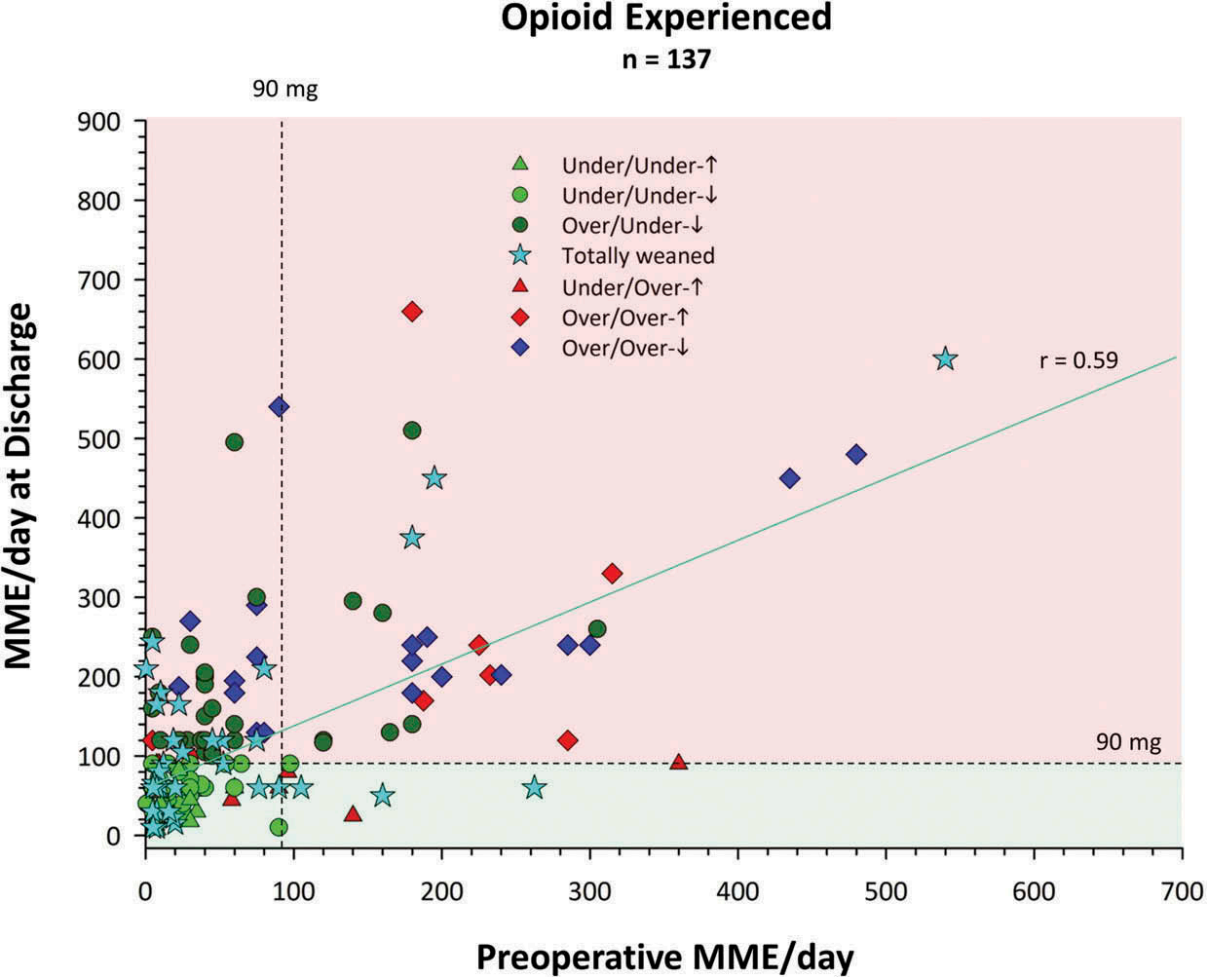
Mean daily opioid use in daily morphine milligram equivalents at hospital discharge shown as a function of daily morphine milligram equivalents preoperatively for opioid-experienced patients. Also shown is the 90 MME/day maximum dose recommended by the U.S. and Canadian opioid guidelines (dashed lines). Legend as in Figure 1.

Figure 1 presents a more fine-grained picture of opioid use for the two groups, showing the daily morphine milligram equivalents dose at hospital discharge after surgery and at the last TPS visit an average of 6 months later based on the 2016 U.S.^69^ and 2017 Canadian^70^ opioid guideline recommendations not to exceed a maximum of 90 MME/day. Not surprising, the scale is doubled for the opioid-experienced group. The two lower quadrants (green shading) show treatment successes and the two upper quadrants (red shading) show failures. The lower left quadrants show patients who were below the recommended 90 MME/day at both time points split into those who were totally weaned by 6 months, those who were below 90 MME/day at discharge and even lower at 6 months, and those whose 6-month dose, though lower than 90 MME/day, was higher than their hospital discharge dose. The lower right quadrant shows patients who were above 90 MME/day at hospital discharge and who were totally weaned by 6 months or had managed to reduce their opioid dose to the recommended dosage or lower. As noted above, these patients represent treatment successes. In contrast, the upper left quadrant shows patients whose discharge dose was 90 MME/day or less but were taking more 6 months later. The upper right quadrant shows patients who were above the 90 MME/day limit on both occasions split into those whose dose was above or below the hospital discharge dose. Figure 2 shows a similar display for the opioid-experienced patients but plotting the hospital discharge dose as a function of the preoperative dose, retaining the symbols and legend from the previous figure depicting the 6-month outcome. Thus, the two upper quadrants indicate that there are at least 14 patients (cyan stars) whose preoperative and discharge doses were between 90 and 600 MME/day but who nevertheless were able to totally wean off opioids by the last TPS visit. Finally, note that the correlation coefficient for opioid-experienced patients shown in Figure 1 (*r* = 0.46) is lower than that in Figure 2 (*r* = 0.59), providing evidence for the efficacy of TPS treatment, which likely was responsible for reducing the magnitude of the relationship (i.e., breaking the link) between hospital discharge and end of TPS treatment opioid use.

**Figure 3.**
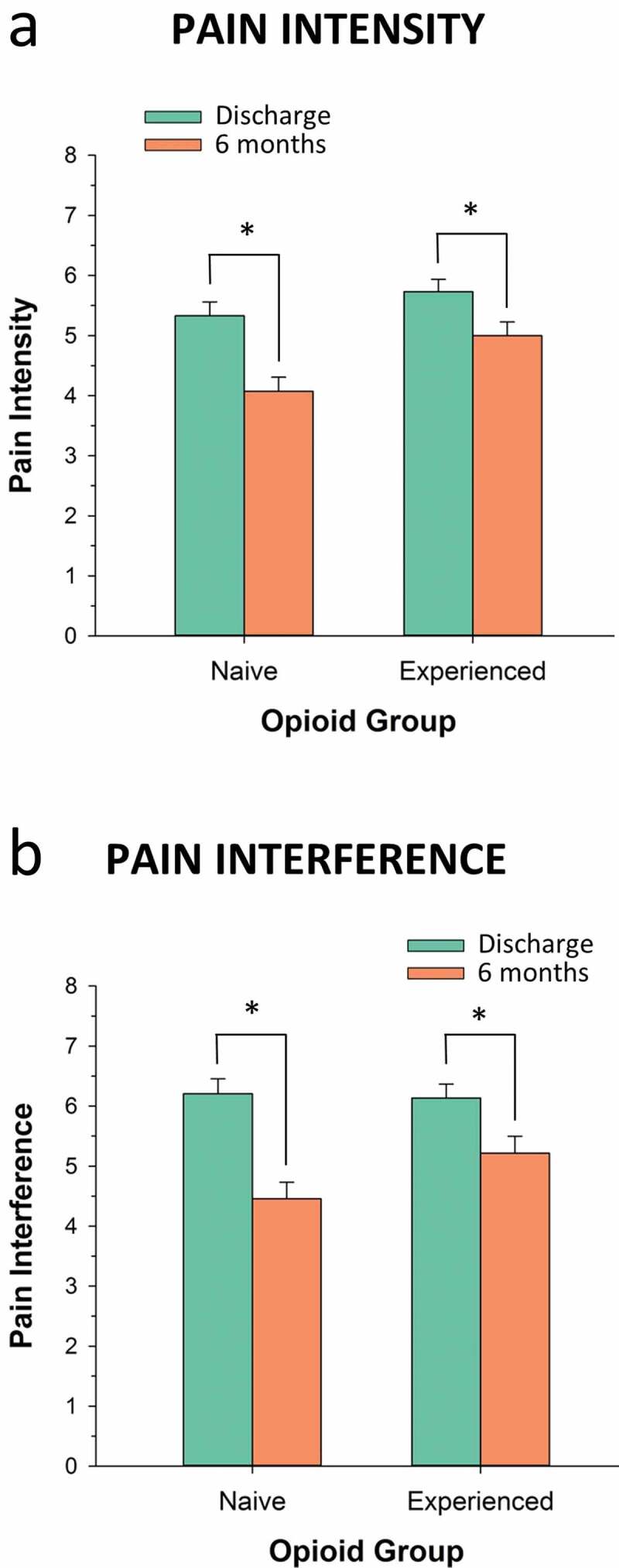
Pain intensity (0–10 Numeric Rating Scale) and pain interference (0–10 Numeric Rating Scale) scores at hospital discharge and at the end of TPS treatment 6 months later shown for opioid-naïve and opioid-experienced patients.  Data from Clarke et al.^65^. *P < 0.009.

Importantly, Brief Pain Inventory (BPI) pain intensity and interference scores at the last TPS visit were significantly lower than their respective post–hospital discharge scores (pre-TPS treatment) in both opioid-naïve and opioid-experienced groups, with the former group reporting larger improvements in pain interference than the latter group (Figure 3). These results indicate that the significant reduction in opioid use is not occurring at the expense of pain and pain-related interference.

As noted above, 19% percent of opioid-experienced patients increased opioid use from hospital discharge to the 6-month time point. This subgroup is clearly of interest because these patients comprise our treatment failures, having faced the greatest challenge weaning from opioids. When compared with opioid-experienced patients who successfully reduced opioid consumption, this subgroup was more likely to be male, an organ transplant patient, and to have been diagnosed with a mental health disorder. For the entire sample of opioid-experienced patients, the predictors of opioid dose reduction from hospital discharge to the last TPS visit included lower pain catastrophizing scores, lower prevalence of neuropathic pain, and a negative history of recreational drug use. In contrast, the only predictor of dose reduction for opioid-naïve patients was the daily morphine milligram equivalent at hospital discharge.^65^

To our knowledge, this is the first study to report detailed data on opioid weaning rates and opioid doses in patients receiving comprehensive, targeted care for postsurgical pain and opioid use in the months after surgery. A multidisciplinary Acute Pain Service outpatient clinic similar to the TPS reported on a sample of 200 patients after surgery.^71^ Fifty-four percent and 32% were discharged from hospital on “weak” and “strong” opioids, respectively. At the end of the outpatient program 3 months later, 20% and 6% of patients were taking weak and strong opioids, respectively. Information on preoperative opioid use and daily morphine milligram equivalent doses before and after surgery were not reported, making it difficult to compare these findings with the TPS results presented above.

## Conclusions

To date, the major known risk factors for CPSP are perioperative pain, opioid use, and negative affect, including depression, anxiety, pain catastrophizing, and posttraumatic stress disorder–like symptoms. The Toronto General Hospital TPS was specially developed to target patients at high risk of developing CPSP based on the above risk factors. Patients are identified early and provided comprehensive care by a multidisciplinary team consisting of pain physicians, advanced practice nurses, psychologists, and physical therapists. Preliminary results from two nonrandomized controlled trials indicate that the TPS effectively reduces pain intensity, pain-related interference, pain catastrophizing, symptoms of anxiety and depression, and opioid use. Almost half of opioid-naïve patients and one in four opioid-experienced patients were opioid free by the 6-month point. A multicenter randomized controlled trial of the TPS in several Ontario hospitals is currently ongoing.
